# Expression of concern: Homonuclear bond activation using a stable *N*,*N*′-diamidocarbene

**DOI:** 10.1039/c4sc90048k

**Published:** 2014-11-25

**Authors:** Robert Eagling

**Affiliations:** a Royal Society of Chemistry , Thomas Graham House, Science Park, Milton Road , Cambridge , CB4 0WF , UK . Email: CHEMICALSCIENCE-RSC@rsc.org

## Abstract

Expression of concern for ‘Homonuclear bond activation using a stable *N*,*N*′-diamidocarbene’ by Kelly M. Wiggins, Jonathan P. Moerdyk and Christopher W. Bielawski, *Chem. Sci.*, 2012, **3**, 2986–2992.



## 

The Royal Society of Chemistry has been contacted by the corresponding author of this article and the Research Integrity Officer at The University of Texas at Austin regarding concerns of scientific misconduct affecting this article. The Research Integrity Officer has informed us that an investigation to ascertain the validity of the work reported has found that scientific misconduct has taken place.

*Chemical Science* is publishing this expression of concern in order to alert our readers that we are presently unsure of the reliability of the data reported in the article. We have contacted the Research Integrity Officer to request more details regarding the scientific misconduct determined in their investigation, in order to determine the appropriate course of action.

An expression of concern will continue to be associated with the article until we receive further information from the Research Integrity Officer on this matter.

Robert Eagling,

25th November 2014,

Executive Editor, *Chemical Science*

